# Quantitative correlation between transcriptional levels of ER chaperone, peroximal protein and FVIII productivity in human Hek-293 cell line

**DOI:** 10.1186/2193-1801-2-328

**Published:** 2013-07-18

**Authors:** Evandra Strazza Rodrigues, Virgínia Picanço-Castro, Marta Regina Espanhol, Luiz Alberto Martins de Andrade, Patricia Vianna Bonini Palma, Simone Kashima, Aparecida Maria Fontes, Dimas Tadeu Covas

**Affiliations:** Hemotherapy Center of Ribeirão Preto, Faculty of Medicine in Ribeirão Preto-FMRP, University of São Paulo-USP, São Paulo, Brazil; Faculty of Medicine in Ribeirão Preto-FMRP, University of São Paulo-USP, São Paulo, Brazil; Faculty of Pharmaceutical Sciences in Ribeirão Preto-FCFRP, University of São Paulo-USP, São Paulo, Brazil; Hemocentro de Ribeirão Preto, National Institute of Science and Technology in Stem Cell and Cell Therapy, Avenida Tenente Catão Roxo, 2501 – Ribeirão Preto, São Paulo, 14051-140 Brazil

**Keywords:** ER BIP chaperone, Factor VIII, Hemophilia A, PAHX gene expression and Recombinant Factor VIII∆B

## Abstract

Hek-293 cell line presents good production platform for recombinant therapeutic proteins, however little is known about the components that contribute to the cellular control of recombinant protein production. In this study, we generated a Hek-293 producing recombinant factor VIII (FVIII) and we evaluated the immunoglobulin-binding protein (BiP) and phytanoil-CoA α-hydroxylase (PAHX) expression levels which are known for diminishing FVIII production. Our analyses showed that the recombinant cell population expresses 3.1 ± 1.4 fold of BIP mRNA (P = 0.0054) and 97.8 ± 0.5 fold of PAHX mRNA (P = 0.0016) compared to nontransduced cells. The amount of these proteins was inversely correlated to the secreted FVIII. In conclusion, BIP and PAHX expression are augmented in human cells producing FVIII and they antagonize the amount of therapeutic factor VIII in the cell culture.

**Electronic supplementary material:**

The online version of this article (doi:10.1186/2193-1801-2-328) contains supplementary material, which is available to authorized users.

## Introduction

Hemophilia A is a hemorrhagic disease caused by insufficient levels or complete absence of functional coagulation factor VIII (FVIII) in the circulation. Hemophilic patients show spontaneous bleeding episodes primarily in soft tissues and joints. This clinical condition can also cause potentially life-threatening hemorrhages in muscles, internal organs and central nervous system (Ghosh 2004; Zago et al. 1987; Antunes et al. 2003).

The current treatment of hemophilic patients consists of prophylactic or therapeutic replacement of the deficient factor by intravenous infusions of FVIII plasma-derived concentrates or recombinant FVIII (rFVIII). Increasingly, the reposition therapy with FVIII concentrates from plasma is substituted by rFVIII produced by mammalian cells culture (Lee 1999; Neidhardt et al. 2005). This source of FVIII brought important therapeutic improvements due to the advantage over the safety of recombinant products compared to those that are plasma-derived. During the 80s, plasma derived products were connected to the spread of HIV and/or hepatitis viruses among thousands of hemophilic patients (Stanley 2006).

Recombinant coagulation factor products are already available in the market for 20 years. So far, FVIII recombinant proteins are produced in murine cells CHO or BHK (Baxter 2010; HealthCare 2009; Wyeth Pharma Pfizer 2007). Although these products have proved to be very similar to the FVIII derived from plasma, recombinant FVIII shows a pattern of nonhuman post-translation modifications (PTM).

FVIII is one of the largest and most complex protein in the market and is produced by recombinant DNA technology (Jiang et al. 2002). This factor is submitted to multiple PTM, particularly glycosylation. Incorrect reproduction of these PTM in a nonhuman expression system may trigger immune reactions and lead to the formation of inhibitors against FVIII. Thus it will be essential to find human cell lines to produce recombinant FVIII with the same glycosylation pattern as the plasma protein. The advantages of using human cell-based expression systems is the high similarity of PTM and the absence of antigenic residues that commonly are present in rFVIII from licensed hamster cell-lines like as cited above (Durocher and Butler 2009). Some studies have associated human HEK 293 cell with a promising potential to rFVIII production in large-scale, using serum-free suspension technology (Picanço et al. 2007; Swiech et al. 2011).

The HEK 293 human embryonic kidney cell line is more often used by the scientific community for the production of recombinant proteins. This strain has rapid growth and it is highly susceptible to transfection and viral transduction (Durocher et al. 2002; Thomas and Smart 2005). Furthermore, there is a commercial adapted version of this strain suspension (Free Style 293-F Cells) which allows the production of large-scale recombinant proteins (Invitrogen Corporation 2007).

Although many PTM are required to obtain a active and non immunogenic FVIII, recombinant FVIII interacts with a number proteins, including calreticulin, calnexin, and immunoglobulin-binding protein (BIP), thus a significant proportion of the FVIII molecule is retained within ER, thereby limiting the transportation of FVIII to the Golgi apparatus. Previous studies showed that the over-expression of BIP inhibits FVIII secretion (Morris et al. 1997) and the antisense RNA expression for BIP reduces BIP levels and improves FVIII secretion (Brown et al. 2011). Another FVIII binding protein, correlated negatively with FVIII expression, is phytanoyl-CoA α-hydroxylase (PAHX). This human protein is a peroxisomal enzyme that not only diminishes FVIII secretion into culture media, but also changes FVIII-A2 intracellular distribution (Chen et al. 2001). Interactions between FVIII and these proteins may limit FVIII productive secretion. Therefore, the strategy for overcoming such limitations may be known the alterations of endoplasmic reticulum (ER) chaperones and peroxisomal components that might contribute to the cellular control of recombinant protein production.

In this study, we evaluated the levels of BIP and PAHX in clones producing FVIII with a partially deleted B-domain (rFVIIIΔB). We found that even using a human cell line with proper machinery to produce FVIII, when it is forced to express high levels of recombinant FVIII, it generates a cellular stress and increases the production of PAHX and BIP that retains part of the FVIII production.

## Material and methods

### Construction of recombinant retroviral vector

Initially, the DNA fragments of 2.29 kb and 2.09 kb referring to the heavy and the light chain of human coagulation FVIII were obtained by polymerase chain reaction (PCR) using the forward 5’- CCG CTC GAG ACC ATG CAA ATA GAG CTC TCC ACC – 3’ and 5’-AGC TTT GTT TAA ACT GAA TTC TGG GAG AAG CTT C-3’ and reverse primers 5’- AGC TTT GTT TAA AC CAA AAC CCA CCA GTC TTG AAA CG -3’ and 5’- ATA AGA ATG CGG CCG CTC AGT AGA GGT CCT GTG CC -3’ respectively. As a positive control, plasmid pSP64 - VIII (American Type Culture Collection- ATCC) was used containing full cDNA the FVIII.

Each amplified DNA fragment was further cloned into pCR^®^2.1-TOPO vector **–** (*Invitrogen, Carisbad, CA, USA*) generating HC_PCR 2.1^+^ (vector containing FVIII heavy chain) and LC_PCR 2.1^+^ (vector containing FVIII light chain). The light chain presents into LC_PCR 2.1 was isolated by restriction endonucleases using the sites *NotI* and *PmeI* to clone into the vector that contains heavy chain (LC_PCR 2.1^+^) previously cut with the same endonucleases. We confirmed sequences of the FVIII light and heavy chain as well as the junction region (53 aas N-terminal of B domain and 12 aas C-terminal B domain) in rFVIII/HC + LC-PCR 2.1^+^ construct by DNA sequencing (*ABI Prism™ DNA Sequencer, Applied Biosystems, CA, USA*). The fragments related to FVIII light and heavy chains with partially deleted B-domain were isolated using restriction *NotI* and *XhoI* endonuclease sites for cloning into pBMN-I-GFP vector previously digested with same enzymes creating BMN-FVIIIΔB-I-GFP. The pBMN-I-GFP vector, which expresses GFP and contains IRES *(internal ribosome entry site)*, was kindly provided by Dr. Garry P. Nolan (NIH, EUA). This construction formed NCBI NM_000132 alignment, which indicated that the constructions were in frame. The retroviral BMN-FVIIIΔB-I-GFP vector, that expresses the partially deleted B-domain FVIII, was used to generate recombinant cell lines.

### PCR

PCR was performed with specific primers for the FVIII heavy and light chains ( 5’- CTT GGA CAG TTT CTA CTG - 3’, 5’- GAC GGA CAT CAG TGA TTC -3’, 5’- GGT CTT CTT TGG CAA TG - 3’, 5’- GGT GTG AAG GAG TCT TG -3’). Reactions were performed in 25 μl of reaction volume containing 100 ng of DNA, 1.0 U of Taq DNA polymerase (*Invitrogen, Carisbad, CA, USA* ), 50 mM KCl, 20 mM Tris–HCl, pH 8.3, 1.5 mM MgCl_2_, 0,2 mM each deoxynucleotide triphosphate (dNTP), and 0.3 pmol of each specific primer. Thermocycling was performed in the GeneAmp PCR system 9700 (*Perkin-Elmer/Cetus, Norwalk, CT*). PCR cycling conditions consisted of an initial cycle of 2 minutes at 94°C, followed by 35 cycles of 40 seconds at 94°C, 1 minute at 56 C, 2.3 minutes at 72°C, and a final extension at 72°C for 10 minutes. The amplified products were analyzed by 1% agarose gel electrophoresis followed by ethidium bromide staining.

### DNA sequencing

DNA sequencing was performed using *ABI Prism*^*®*^*Big Dye*™ *Terminator Cycle Sequencing Ready Reaction Kit* (*Applied Biosystems, Foster City, CA, USA*), according to the manufacturer’s instructions, and electrophoresed, directly with an automated apparatus *ABI Prism 377 DNA Sequencer* (*Applied Biosystems, Foster City, CA, USA*). The electropherograms were analyzed by Sequence Analyzer software *ABI Analysis Data Collection* version 3.3 (*Applied Biosystems, Foster City, CA, USA*). The obtained sequences were aligned with the reference sequence available on GenBanK (NM_000132) by Sequencher software version 4.05 (*Gene Codes Corp, Ann Arbor, MI, EUA*).

### Cell culture

PG13/LN c8 cell line and human embryonic kidney 293 cells (Hek-293) (*American Type Culture Collection* –ATCC *Manassas, VA*) were maintained in Dulbecco’s modified Eagle’s medium (Gibco-BRL) supplemented with 2 mM L-glutamine (*Gibco-BRL, Gaithersburg, MD, USA*), 3.7 g/l sodium bicarbonate, 2.4 g/l buffer HEPES, 10% fetal calf serum (*HyClone, Logan, UT, USA*), and 100 U penicillin/streptomycin (*Sigma, Grand Island, NY, USA*). All the cell lines were maintained in a humidified incubator under 5% CO_2_ at 37°C.

### Transfection and transduction of cells

The packaging PG13/LN c8 cell line was cultured into 100 mm plates and incubated overnight until 80% confluence. The cells were further transfected with the BMN-FVIIIΔB-I-GFP vector using *LipofectAMINE™* Reagent kit (*Invitrogen, Carisbad, CA, USA*), following manufacturer’s instructions. Four hours after transfection, cells were washed with buffer PBS (1X), reduced volume of fresh culture medium was added and the cells were incubated under 5% CO_2_ in a humidified incubator. The viral supernatant was collected at 24, 48 or 72 hours and used for transduction.

To infect Hek 293 cells, the supernatant with infectious virus, produced by packaging PG13/LN c8 cells after transfection, was daily collected, filtered by 45μm filter (*Millipore, Billerica, MA, USA*), mixed with 5.5 μg/μl of polybrene (*hexadimethrine bromide - Sigma, MA, USA*) and incubated with Hek-293 cells for 16 hours. This procedure was repeated three times.

### Analysis and sorting of cells by fluorescent flow cytometry

After transduction, Hek-293/BMN-FVIIIΔB-I-GFP cells were harvested, washed in 1X PBS, centrifuged at 335 g, resuspended in buffer 1X PBS and analyzed by flow cytometry (FACsort, BD, San Jose, CA, USA) to quantify the percentage of GFP-positive cells. GFP-positive cells were sorted using a FACS Vantage (Becton Dickinson, San Jose, CA, USA).

### Real-time PCR

RNA was extracted from 5 × 10^6^ cells using Trizol (Invitrogen, Carisbad, CA, USA) and cDNA was synthesized using High Capacity cDNA Reverse Transcription Kit (Applied Biosystems, Foster City, CA, USA) using the following reaction: 2.0 μg RNA, Random Primers 1X, RT Buffer 1X, 10 mM dNTP’s, 0.3μl RNAse OUT and 50U MultiScribe™ MuLV reverse transcriptase in a total volume of 20 μl and incubated at 25°C for 10 min, 37°C for 120 min. SYBR Green real-time PCR was carried out using ABI Prism 7500 Sequence Detection System (Applied Biosystems, Boston, MA) in a total volume of 15 μl containing 2.5 pM each specific primers (Table 1), SYBR Green PCR Master Mix, and 2 μl of cDNA. After initial incubations at 50°C for 2 min and 95 °C for 10 min, 40 cycles of amplification were carried out for 15 s at 95°C followed by 1 min at 60°C. Human glyceraldehyde-3-phosphate dehydrogenase (GAPDH) was used as endogenous control to standardize the amount of applied RNA and to confirm the quality of the isolated RNA. Single-product amplification was confirmed by postmelting curve. Duplicate samples were measured and averaged. Relative FVIII light and heavy chain, BIP chaperone, and phytanoyl-CoA α-hydroxylase (PAHX) transcription levels were measured as expression relative unit. The difference in CT values between the analyzed genes and endogenous control reactions (dCT) was converted into expression relative units by setting GAPDH equal to 10 000 and dividing by 2dCT (Albesiano et al. 2003).

**Table 1:** **Primers used in PCR Real-time analysis**

Primer Name	Primer sequence
P5 BiPH	5’- CCA ACG CCA AGC AAC CAA AG-3’
P3 BiPH	5’- CTT CTC CCC CTC CCT CTT AT-3’
P5 FytCoAH	5’- CTG TCT GGT TGT GCT CCC A-3’
P3 FytCoAH	5’- GCC TTG TTT TCC TCG TAG TCC-3’
P5 CHFVIII	5’- CAC TCT TGA TGG ACC TTG GAC-3’
P3 CHFVIII	5’- TCG TAG TTG GGG TTC CTC TG-3’
P5 CLFVIII	5’- GAT GGG AAG AAG TGG CAG AC-3’
P3 CLFVIII	5’- GTG CAA ACG GAT GTA TCG AGC-3’
P5 GAPDH	5’- GCC TCA AGA TCA TCA GCA ATG C-3’
P3 GAPDH	5’- CAT GGA CTG TGG TCA TGA GTC CT-3’

### Activated partial thromboplastin time (APTT)

*In vitro* recombinant human FVIII activity from supernatants of transduced Hek 293 cells cultured in the presence of inducers of calcium ionophore (A-23187) protein secretion (3 μg/ml), and Phorbol 12-myriastate 13-acetate (PMA) (6 μg/ml) for 24 hours were quantified by measuring the FVIII-dependent generation of thrombin using one stage clotting assay ATTP (*Biomérieux*, Sweden). FVIII deficient plasma was used as substrate, human plasma purified FVIII *Verify* – *Reference plasma* – (*Organon Teknika, Durham*) was used as a positive qualitative standard, and 1,000 mU was defined as 200 ng FVIII/ml or 1U as 100% of activity. For the thrombin activation assay, conditioned medium of transduced Hek 293 cells was diluted (1:5) and incubated at room temperature. After incubation, the FVIII activity was measured by *COAG-A-MATE*^*®*^*XM - Organon Teknika* according to the manufacturer’s specifications.

### Statistical analysis

The normality of the data was examined by Shapiro-wilk test using R (version 3.0) p ≤ 0.05. Then, the data was analyzed applying t-test with Mann–Whitney test or nonparametric correlation (Spearman) test, one-tail using the GraphPad InStat software, version 3.0 for Windows (GraphPad Software, San Diego, CA, USA; http://tools.invitrogen.com/content/sfs/manuals/FreeStyle_293_F_Cells_man.pdf), with the level of significance set at p ≤ 0.05.

## Results

### Construction of the BMN-FVIIIΔB-I-GFP retroviral plasmid

**Figure 1:**
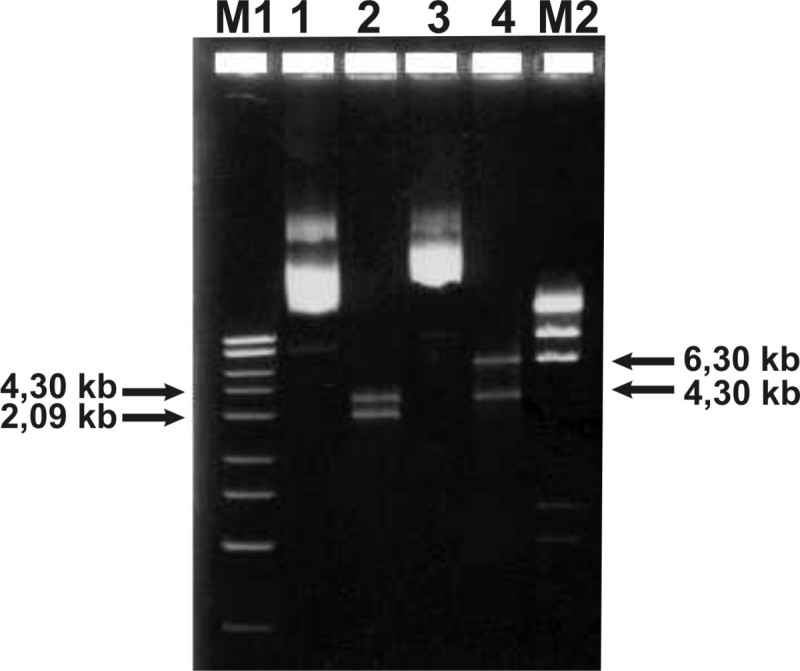
**Cloning FVIIIΔB in pBMN-I-GFP.** FVIII∆B containing FVIII heavy and light chain with B-domain partial deleted was cloned first in pCR2.1-TOPO plasmid and after in pBMN-I-GFP. Agarose gel (1%) stained with ethidium bromide after electrophoresis showing both clones. Lanes 1 and 3: DNA from recombinant clones without restriction enzyme digestion. Lane 2: *Xho* I/*Not* I digested positive FVIII∆B clone in pCR2.1-TOPO plasmid. Lane 4: *Xho* I/*Not* I digested positive FVIII∆B clone in pBMN-I-GFP plasmid. Lanes M: 1 kb DNA ladder and lambda DNA digested with *Hind* III.

To generate the retrovirus bicistronic vector BMN-FVIIIΔB-I-GFP, rFVIIIΔB was amplified by PCR using specific primers to amplify the heavy chain (domain A1, A2 plus 53 aas N-terminal of B domain) and the light chain (domains A3, C1, C2 plus 12 aas C-terminal B domain) of hFVIII. These DNA fragments were joined and generated one common fragment with 4.3 kb DNA. This DNA fragment was first cloned into pCR2.1-TOPO vector (Figure 1: lanes 1, 2) and then into expression vector pBMN-I-GFP (Figure 1: lanes 3, 4). The cloned sequence authenticity was confirmed by DNA sequencing.

After the retroviral transduction, *Hek-293/BMN-FVIIIΔB-I-GFP* cells were sorted by FACS to obtain a cell population with high level of eGFP. Fifteen days later, the flow cytometry analysis showed that all derived population (*Hek-293/BMN-FVIIIΔB-I-GFP 1*–*15*) were GFP positive and presented the proportion of GFP^+^ cells higher than 40% (Table 2).

**Table 2:** **Biological activity of FVIII secreted by** ***Hek-293/BMN-FVIIIΔB-I-GFP***

Sample	% of GFP expression	Activity (%)	IU/mL
Culture medium DMEN	-	1.5	-
S. Cell pop. 01	74.0	14.0	0.1
S. Cell pop. 02	80.0	11.1	0.1
S. Cell pop. 03	76.5	10.0	0.1
S. Cell pop. 04	72.1	34.6	0.3
S. Cell pop. 05	70.0	41.9	0.4
S. Cell pop. 06	70.5	21.7	0.2
S. Cell pop. 07	68.1	1.9	0.0
S. Cell pop. 08	63.8	2.2	0.0
S. Cell pop. 09	44.3	2.9	0.0
S. Cell pop. 10	43.4	20.1	0.2
S. Cell pop. 11	44.9	4.1	0.0
S. Cell pop. 12	48.2	75.8	0.7
S. Cell pop. 13	53.6	18.5	0.1
S. Cell pop. 14	62.5	47.9	0.4
S. Cell pop. 15	48.6	37.1	0.3

Analysis of biological activity of FVIII secreted by *Hek-293/BMN-FVIIIΔB-I-GFP* using TTPA assay. *S* Supernatant, *Cell pop* Cell population, *Hek-293* human embryonic kidney epithelial cells.

### Correlation between percentage of GFP^+^ cells and FVIII mRNA expression level

**Figure 2:**
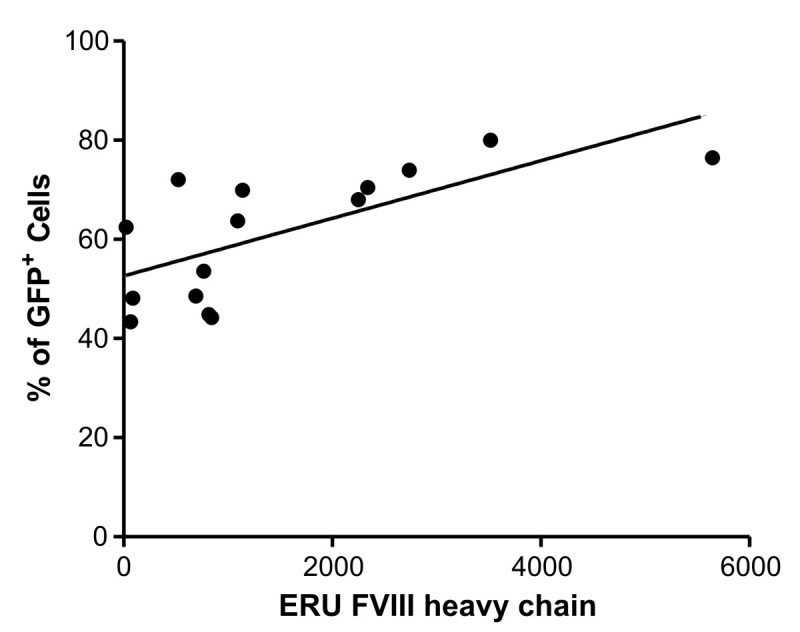
**Correlation between mRNA FVIII and GFP expression in Hek-293/BMN-FVIIIΔB-I-GFP cell population.** Correlation analysis between mRNA of FVIII heavy chain and the percentage of GFP positive cells (*p =* 0.0015, *r =* 0.71) n = 15. Data are reported as expression relative units (ERU). We used nonparametric correlation (Spearman) test, one-tail and data were considered statistically significant (p < 0.05).

We evaluated the expression of the light and heavy chain of FVIII by semi-quantitative reverse transcription-PCR in fifteen isolated cell population and compared with the percentage of GFP positive cells to assess the GFP efficiency as a gene marker to select rFVIII cell producers. The GAPDH gene was used as endogenous control, showing a homogeneous profile expression among the studied samples (SD = 0,8). As expected, we found a positive correlation between the mRNA expression of FVIII and the percentage of GFP^+^ cells selected in all *Hek-293/BMN-FVIIIΔB-I-GFP* clones (*r* = 0.71, *p* = 0.0015)(Figure 2).

### mRNA expression of BIP and PAHX in the Hek-293/BMN-FVIIIΔB-I-GFP cell population

**Figure 3:**
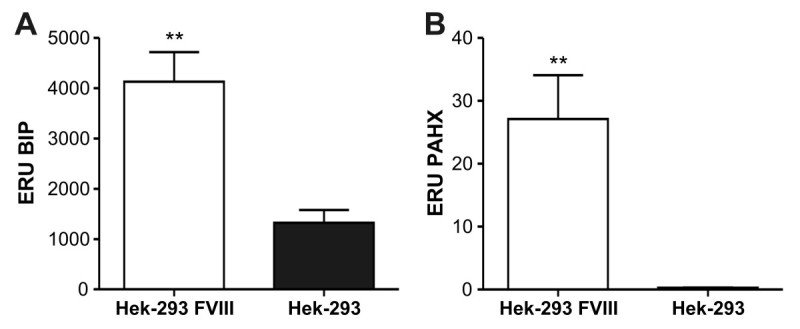
**Gene expression of mRNA of BIP and PAHX among Hek-293/BMN-FVIIIΔB-I-GFP cell population. A** and **B)** Gene expression of mRNA of BIP and PAHX in Hek-293/BMN-FVIII∆B-I-GFP cells and Hek-293 non-transfected cells. Data are reported as expression relative units (ERU) and are represented as the means ± SEM. We used nonparametric t-test (Mann Whitney), one-tail and data were considered statistically significant (p < 0.05). The asterisks denote significant differences compared to non-transfected Hek-293 (** p < 0.01).

Considering that rFVIII expression in cell lines can be modulated by proteins that interact with the FVIII molecule and limit mRNA translation, transportation into the Golgi complex and secretion, we evaluated the mRNA expression level of two proteins that are known for interacting with FVIII (*BIP* and *PAHX*). The real-time PCR analysis of the 15 selected population showed an increase of 3.1 ± 1.4 fold of BIP mRNA expression on *Hek-293/BMN-FVIIIΔB-I-GFP* cell population when compared to non-transfected Hek-293 cells (*p =* 0.0054) (Figure 3A). We also observed an induction of 97.8 ± 0.5 fold expression of mRNA PAHX in the cell population producers of rFVIII (*p =* 0.0016) (Figure 3B). These results indicate that the overexpression of FVIII mRNA induces BIP and PAHX expression because of the cellular stress created by the recombinant protein.

### Protein secretion of FVIII and the mRNA expression of BIP and PAHX

To investigate the impact of the expression FVIII-ligand proteins in the rFVIII secretion, we verified the correlation between expression (BIP and PAHX) and biological activity of the FVIII.

**Figure 4:**
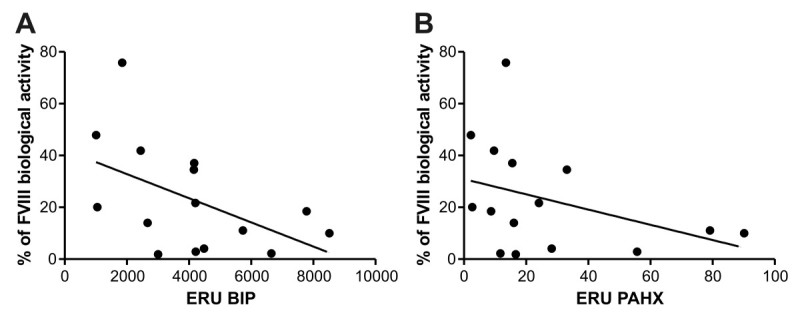
**Comparison between transcription levels BIP and PAHX and FVIII secretion. A)** Correlation analysis between mRNA of BIP and the percentage of FVIII biological activity (*p =* 0.0090, *r = -* 0.60). **B)** Correlation analysis between mRNA of PAHX and the percentage of FVIII biological activity (*p =* 0.042, *r = -* 0.46). Data are reported as expression relative units (ERU). We used nonparametric correlation (Spearman) test, one-tail and data were considered statistically significant (p < 0.05).

Among the FVIII selected population, we observe an inversely proportional correlation between mRNA BIP expression and the value of the biological activity (*p =* 0.009 *r = -* 0.60) (Figure 4A). Similar results were obtained when we compared FVIII secretion to the expression levels of mRNA of PAHX, showing a negative correlation (*p =* 0.042 *r = -* 0.46) (Figure 4B).

## Discussion

In this work, we used retroviral system for the expression of FVIII recombinant molecule that contains light and heavy chains of the protein and a small segment of the B domain. Native full-length human FVIII consists of 2332 amino acid residues divided into the following domains: A1-a1-A2-a2-B-a3-A3-C1-C2; the heavy chain comprises the domains A1-a1-A2-a2-B and light chain a3-A3-C1-C2. The chains as linked by a divalent metal ion (Shen et al. 2008; Lenting et al. 2010). Previous reports in the literature showed that B domain does not play an important role in the biological activity protein and can be removed without losing its function for blood coagulation (Toole et al. 1986). rFVIII constructs with partial deleted B domain have shown the presence of biologically active FVIII in the supernatant of different cell cultures (Meulien et al. 1988; Lind et al. 1995; Herlitschka et al. 1998; Burton et al. 1999). Another advantage is the removal of the B-domain is that it allows nano-filtration of the recombinant protein enhancing purity and safety. The B-domain is highly glycosylated, but it is not essential for the pro-coagulant function of the protein. The B domain may be important for intracellular processing and trafficking during biosynthesis (Pipe 2009). The recombinant products in the market are produced in murine cell lines that have in their structure the antigenic carbohydrate epitopes α-Gal and Neu5Gc, which are absent in human-FVIII. The FVIII production in a human cell line avoids the potential incorporation of non-human glycan epitopes (Kannicht et al. 2013).

Human cell lines for the production of FVIII are highlighted as an improvement in the treatment of Hemophilia A. These cells have proper machinery for PTM and secretion of FVIII. In this work, we evaluated if the forced expression of FVIII in a human cell line (Hek 293) can alter the expression of FVIII-ligand proteins (BIP e PAHX) what can influence FVIII production. Our work showed that the transcription levels of mRNA of BIP and PAHX in the *Hek-293/BMN-FVIIIΔB-I-GFP* were increased in relation to the non-transfected cell. Similar results have been described by (Becker et al. 2004) who demonstrated that FVIII mRNA expression induce a rather moderate increase of BIP transcription in COS cells transfected with rFVIII full length and B-domain deleted, reflecting the cellular stress created by the recombinant protein.

The mRNA expression levels of BIP and PAHX were reversely proportional to the secretion level of biologically active FVIII. BIP protein is supposed to interact with A1 domain of FVIII and decrease FVIII secretion (Marquette et al. 1995; Dorner et al. 1992; Dorner and Kaufman 1994). To solve this problem, other authors (Miao et al. 2004; Srour et al. 2008) suggest the construction of rFVIII molecules with mutations within the A1 domain to reduce the interaction with BIP chaperone protein (Miao et al. 2004), or the addition of a glycosylation site in the N terminal region of FVIII protein (Srour et al. 2008).

PAHX mRNA has been found in specific tissues like liver and kidney. As Hek-293 is a kidney derived cell line, we evaluated if PAHX expression could alter FVIII secretion. Our work shows that Hek 293 cell population with the highest FVIII secretion levels presented low expression of mRNA PAHX. Chen *et al.* showed that PAHX overexpression in BHK cells producing rFVIII is related to 70% decrease of FVIII protein expression, according to quantification of bands on the immunoblotting (Chen et al. 2001).

Many strategies have already been applied as an attempt to improve FVIII secretion and to diminish the costs of rFVIII production. The production of recombinant FVIII in murine cells should be replaced by the production in human cells. However, it is necessary to evaluate the stress caused by the high expression of exogenous FVIII in human cells and develop strategies for inhibiting and/or silencing proteins as BIP and PAHX. Our work suggests the screening of BIP and PAHX mRNA of FVIII Hek 293cell producers as a new perspective to improve and facilitate FVIII production.

## Authors’ original submitted files for images

Below are the links to the authors’ original submitted files for images. Authors’ original file for figure 1 Authors’ original file for figure 2 Authors’ original file for figure 3 Authors’ original file for figure 4
