# Supplementary figures and images for: Quantitative correlation between transcriptional levels of ER chaperone, peroximal protein and FVIII productivity in human Hek-293 cell line
Source: Springerplus. 2013 Jul 18;2(1):328. doi: 10.1186/2193-1801-2-328 (PMC4255388; doi:10.1186/2193-1801-2-328)

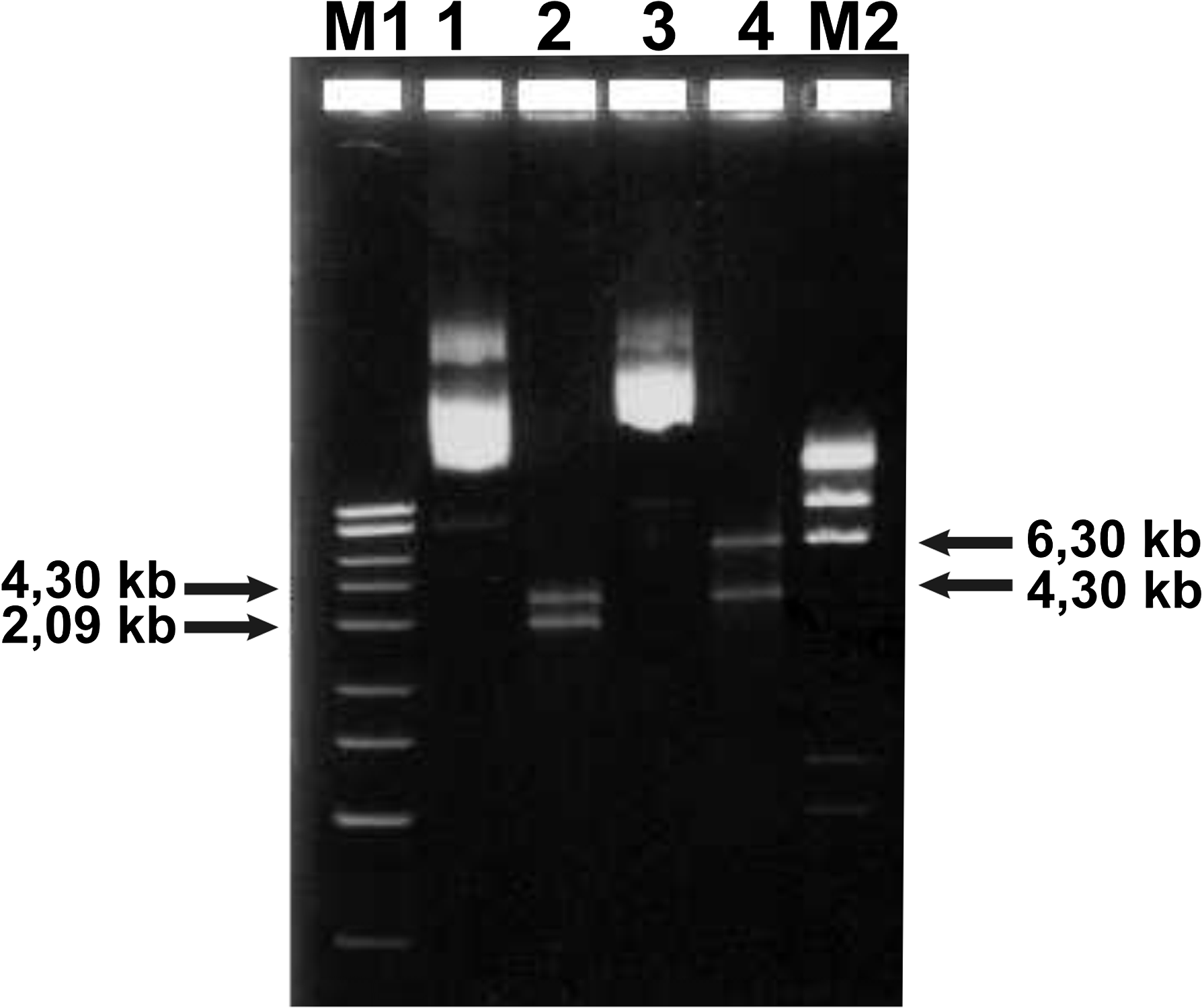

Supplement: Supplementary file 1 — Authors’ original file for figure 1 [file 40064_2013_442_MOESM1_ESM.tif]

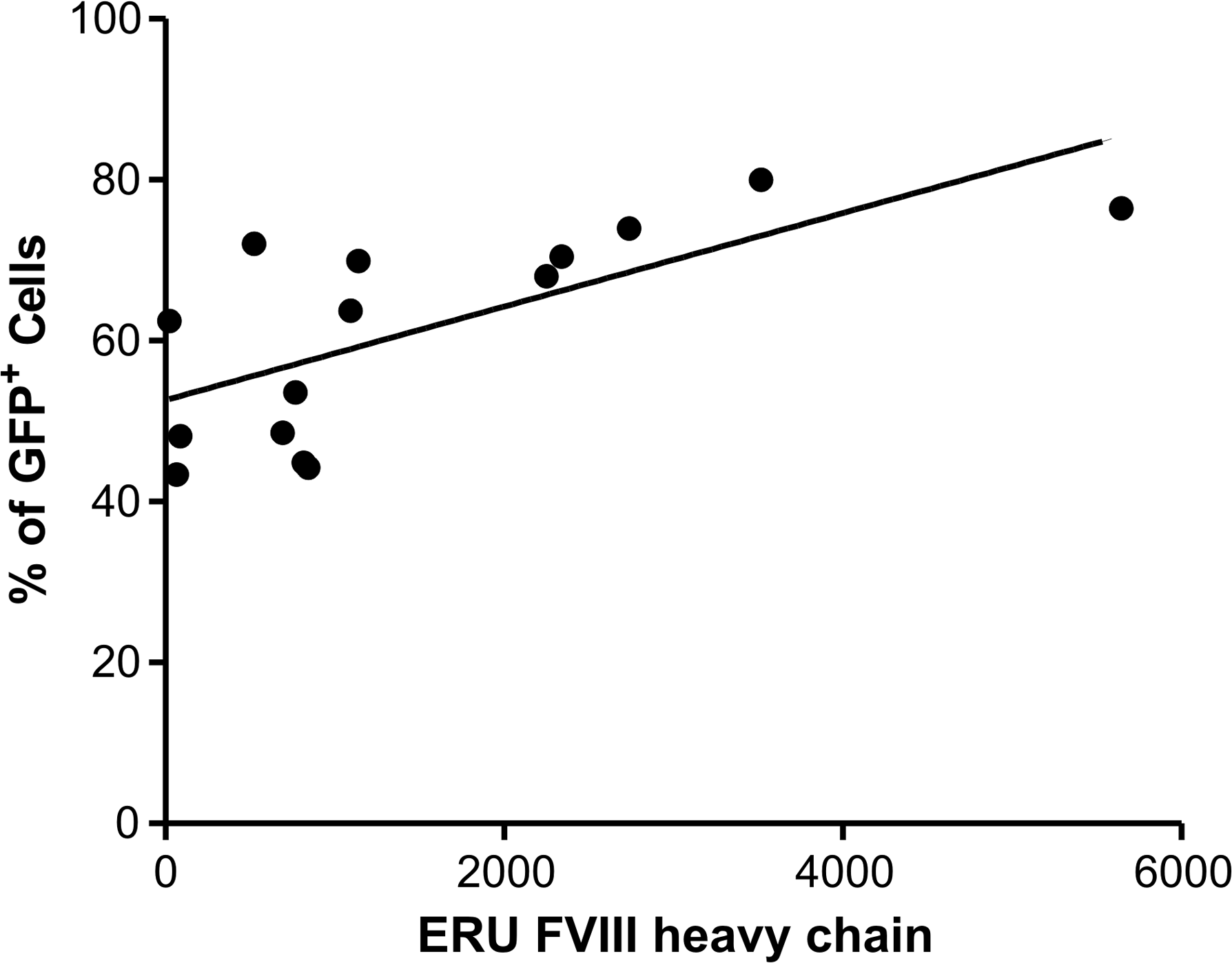

Supplement: Supplementary file 2 — Authors’ original file for figure 2 [file 40064_2013_442_MOESM2_ESM.tif]

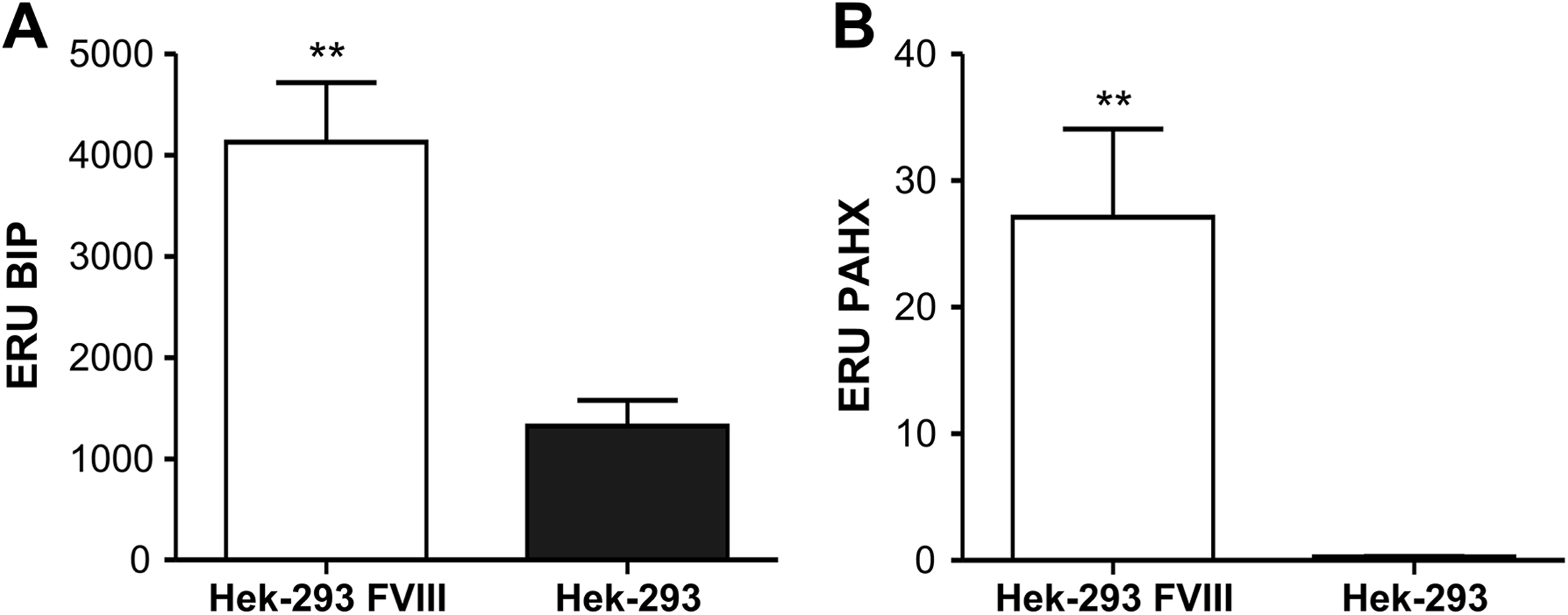

Supplement: Supplementary file 3 — Authors’ original file for figure 3 [file 40064_2013_442_MOESM3_ESM.tif]

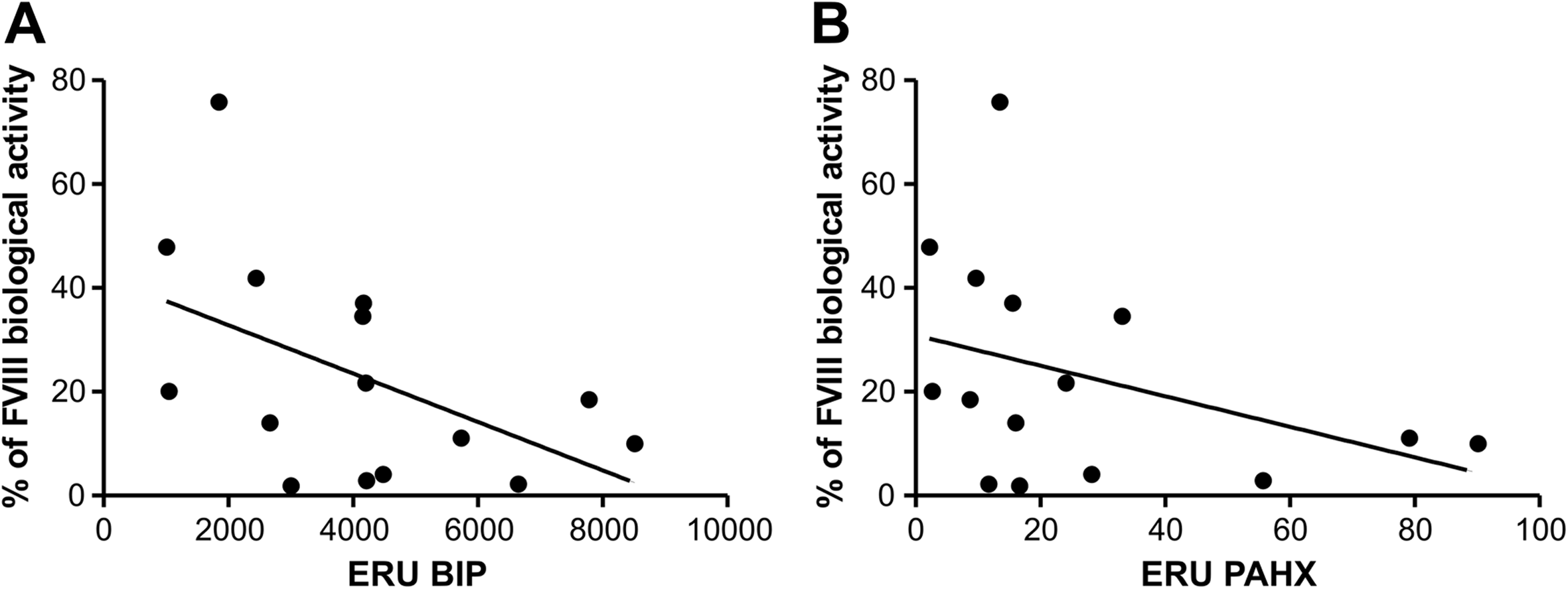

Supplement: Supplementary file 4 — Authors’ original file for figure 4 [file 40064_2013_442_MOESM4_ESM.tif]
